# Predicting intraoperative major blood loss in microsurgery for brain arteriovenous malformations

**DOI:** 10.3389/fmed.2024.1446088

**Published:** 2024-08-07

**Authors:** Jichun Shi, Shuangxiang Xu, Yu Feng, Wei Wei, Yichun Zou, Wenping Xiong, Wenyuan Zhao, Tingbao Zhang, Hao Peng, Jincao Chen

**Affiliations:** ^1^Department of Neurosurgery, Zhongnan Hospital of Wuhan University, Wuhan, Hubei, China; ^2^Department of Neurosurgery, Hainan Affiliated Hospital of Hainan Medical University (Hainan General Hospital), Haikou, Hainan, China; ^3^Department of Neurosurgery, The Second People’s Hospital of Hainan Province, Haikou, Hainan, China

**Keywords:** brain arteriovenous malformations, microsurgery, blood loss, predictive model, bone-wax coated bipolar electrocoagulation

## Abstract

**Objective:**

Intraoperative blood loss poses a great challenge for brain arteriovenous malformation (AVM) microsurgery, although systematic researches are still lacking. This study aimed to identify factors predicting intraoperative major blood loss in brain AVM microsurgery and to investigate its impact on patient outcome. To deal with the fierce bleeding, we introduced a modified hemostatic method, bone-wax (BW) coated bipolar electrocoagulation.

**Methods:**

The authors retrospectively analyzed the clinical data of 131 patients (50/81 in intraoperative major/non-major blood loss cohort) with brain AVMs who underwent microsurgery in our center during the period between January 2018 and April 2023. According to previous studies, major blood loss was defined as blood loss of at least 1,000 mL. The accuracy and objectivity of our grouping methodology were validated by comparing the hemoglobin mass loss, hematocrit loss and factors associated with intraoperative bleeding. Potential clinical and radiological predictors for intraoperative major blood loss were evaluated using a multivariate stepwise logistic regression. And outcomes of patients in the two cohorts were also compared. At last, the performance of BW coated bipolar electrocoagulation in brain AVM microsurgery was illustrated by the case presentation, histological staining and transmission electron microscopy of the coagulated nidus vessels.

**Results:**

Hemoglobin mass loss, hematocrit loss and factors associated with intraoperative bleeding were significant different between the two cohorts. five independent factors predicting intraoperative major blood loss were identified: (1) clinical manifestations; (2,3) location and size of the nidus; (4) deep venous drainage; and (5) the number of draining veins. And the intraoperative major blood loss can not only adversely affect the surgical progression, but also predict poor perioperative outcomes for patients. Regarding the application of BW coated bipolar electrocoagulation, we found the novel hemostatic method exerted efficient hemostatic effect and reduced the damage to the vascular structure in brain AVM microsurgery.

**Conclusion:**

This study proposed a nomogram for neurosurgeons to predict intraoperative major blood loss in brain AVM microsurgery preoperatively. And intraoperative major blood loss is associated with poor patient outcomes. In addition, BW coated bipolar electrocoagulation, can be applied to control ferocious bleeding during brain AVM microsurgery, which still remains further researches.

## 1 Introduction

Brain arteriovenous malformations (AVMs) is a relatively rare cerebrovascular disease, which is characterized by the dysplastic vascular tangles (nidus) that form direct connections between feeding arteries and draining veins (1). Overall, brain AVM accounts for 25% of hemorrhagic strokes in adults < 50 years of age (2). And the primary objective of brain AVM management is the prevention of future hemorrhagic episodes. Apart from conservative observation, the modalities of brain AVMs treatment include microsurgical resection, endovascular embolization, and stereotactic radiosurgery, alone or in any combination (1). Among these modalities, microsurgery has advantages of the highest instant cure rate and the lowest risk of future hemorrhage (3, 4). Nevertheless, the intrinsic characteristics of AVMs, include the high flow and low resistance hemodynamics (5, 6), the aberrant vascular structure (7, 8), and similarities in feeding arteries and draining veins (9), make the fierce bleeding during microsurgical resection a great challenge. Intraoperative major blood loss can not only adversely affect the surgical progression, but also predict poor prognosis for patients (10). Currently, there is still lacking systematic studies on the topic of intraoperative blood loss in brain AVMs microsurgery, and some studies showed mixed results (3, 11).

In the current study, we aimed to identify the contributing and independent predictive factors of intraoperative major blood loss in brain AVMs microsurgery, which can be helpful for neurosurgeons to predict the risk of intraoperative major blood loss in advance and develop the reasonable surgical strategy. The impact of intraoperative major blood loss on patient outcome was also addressed. Notably, our previous work has developed a modified electrocoagulation hemostatic technique, bone-wax (BW) coated bipolar electrocoagulation, which can provide a convenient, cost-efficient, safer, and more efficient way for intraoperative hemostasis (12). To deal with the fierce bleeding, we presented a preliminary overview of the use of this novel hemostatic method in microsurgery of brain AVMs.

## 2 Materials and methods

### 2.1 Study population

**FIGURE 1:**
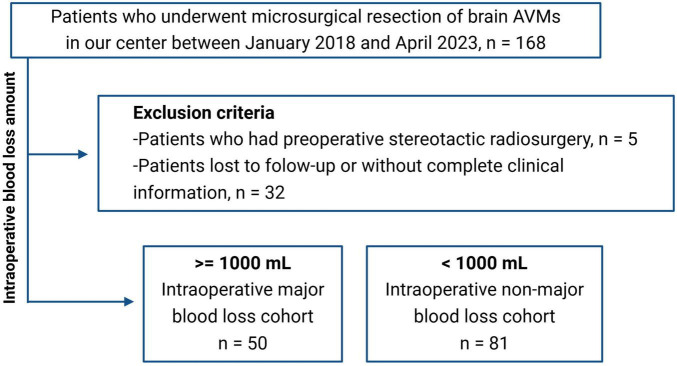
Flow chart of patient selection.

The flow chart of the patient selection process is presented in Figure 1. The authors conducted a retrospective study on data for 168 patients treated in Zhongnan Hospital of Wuhan University who underwent microsurgical resection of brain AVMs during the period between January 2018 and April 2023. Patients who had preoperative stereotactic radiosurgery (*n* = 5) were excluded. Patients lost to follow-up or without complete clinical information (*n* = 32) were excluded. A total of 131 patients were included in this study. This retrospective study was approved by the local ethics committee of Zhongnan Hospital of Wuhan University (Ethics No. Kelun-2024058K). Due to the low risk of a privacy breach and the retrospective nature of the study, a waiver for patient consent was granted.

The amount of intraoperative blood loss was retrospectively recorded and defined as the estimated blood loss from the anesthesia case record. And according to intraoperative blood loss, the study population was divided into two groups. Similar with the previous study (10), we defined patients with intraoperative blood loss of at least 1,000 mL as the intraoperative major blood loss cohort (*n* = 50), and the other as the intraoperative non-major blood loss cohort (*n* = 81).

### 2.2 Objectivity and accuracy validation of study subgroups

Visual estimation by anesthetists and surgeons is still the mainstay to estimate intraoperative blood loss. However, the objectivity and accuracy of this rough method remain controversial. Previous studies indicated that estimating intraoperative blood loss by hemoglobin (Hb) can be a more accurate method (13, 14). In order to verify the objectivity and accuracy of study subgroups, the relative changes of intraoperative Hb (ΔHb_intraop._) and perioperative Hb (ΔHb_periop._) in major/non-major blood loss cohort was compared. And the resulting formula is summarized as:

$$\Delta {\text{Hb}}_{\text{intraop}.}\left(\%\right)={\left[({\text{Hb}}_{\text{intraop}.\text{max}}–{\text{Hb}}_{\text{intraop}.\text{min}})/{\text{Hb}}_{\text{intraop}.\text{max}}\right]}^{*}\text{ 100}$$

$$\Delta {\text{Hb}}_{\text{periop}.}\left(\%\right)={\left[\left({\text{Hb}}_{\text{preop}.}–{\text{Hb}}_{\text{postop}.}\right)/{\text{Hb}}_{\text{postop}.}\right]}^{*}\text{ 100}$$

Hb_intraop.max_ / Hb_intraop.min_ is the patient’s intraoperative highest / lowest hemoglobin concentration (g/dL) measured intraoperatively. Hb_preop._ / Hb_postop._ is the patient’s intraoperative preoperative / postoperative hemoglobin concentration (g/dL).

According to this way of defining, hematocrit (Hct) relative change is also calculated, including ΔHct_intraop._ and ΔHb_periop._. Besides, other parameters related to intraoperative blood loss were also recorded and compared between the major and non-major blood loss cohorts. These parameters included crystalloid fluid replenishment, colloid fluid replenishment, plasma replenishment, autologous blood intake, suspended red blood cell intake, single donor platelet intake, coagulation factor intake and the intraoperative urine output.

### 2.3 Study variables

Variables included patient demographics, AVM characteristics and surgical characteristics. AVM characteristics included the components of supplemented Spetzler-Martin (SM) grades (age, rupture history, compactness of the AVM nidus, AVM size, venous drainage pattern, and eloquence), nidus location, associated aneurysm, clinical presentation, and angio-architectural characteristics (number of feeding arteries, number of draining veins, maximum diameter of draining veins, and brain AVM flushing time). Brain AVM flushing time (bAFT) was defined as the time interval from the start of visualization to complete non-visualization of the AVM nidus in DSA. For data accuracy, all angio-architectural data were collected by neuro-interventional radiologists. Surgical characteristics included coagulation function, ASA score, surgeon, surgical position, and whether it was an emergency surgery. Data of treatment style (whether or not combined with preoperative endovascular embolization) were also collected.

The modified Rankin Scale (mRS) preoperatively and at last available follow-up was measured for neurological status evaluation. Good outcomes were defined as mRS scores ≤ 2, and poor outcomes were defined as mRS scores > 2. Besides, outcome indicators also included length of surgery, postoperative length of stay, rate of severe surgical complications, and rate of complete lesion resection.

### 2.4 BW coated bipolar electrocoagulation

A detailed description of BW coated bipolar electrocoagulation has been shown in our previous work (12). Briefly, neurosurgeons just need to dip the forceps tips through the elastic mesh into melted BW and take it out. A small amount of BW can be applied evenly to the tips of bipolar electrocoagulation forceps. Electrocoagulation with BW coated bipolar electrocoagulation forceps is known as BW coated bipolar electrocoagulation.

### 2.5 Statistical analysis

All statistical analysis was performed using the open-source R software (version 4.2.1). Two-tailed unpaired Student’s *t*-test and one-way ANOVA were used to compare data between two groups and more than two groups, respectively. Categorical variables were analyzed with the *chi*-square test and Fisher’s exact test. A univariate logistic regression analysis was performed to identify factors associated with the intraoperative major blood loss. To avoid significant factors being omitted, risk factors found on the univariate analysis to have *p*-values < 0.2 were included in a multivariate logistic regression model. The area under the receiver operating characteristic curve (AUC) was calculated, the odds ratios (ORs) and 95% CIs were also calculated. R package used in this study for nomogram, calibration curve and decision curve include “car, rms, pROC, Hmisc, rmda.”

## 3 Results

### 3.1 Objectivity and accuracy of study groupings

**FIGURE 2:**
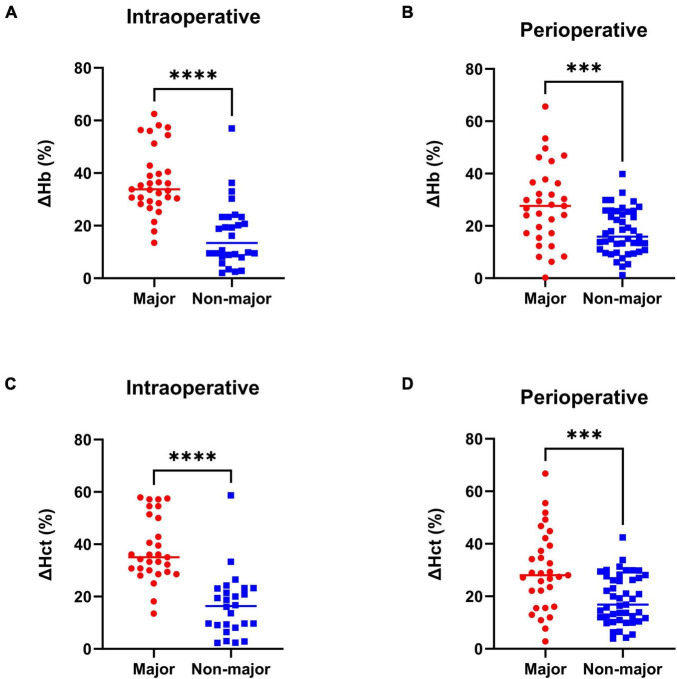
Relative change of Hb and Hct in the major and non-major blood loss cohort. **(A)** Intraoperative Hb relative change. **(B)** Perioperative Hb relative change. **(C)** Intraoperative Hct relative change. **(D)** Perioperative Hct relative change. *n* = 50 in the major blood loss cohort and *n* = 81 in the non-major blood loss group. Two-tailed unpaired *t*-test, ***p < 0.001, ****p < 0.0001. Forest plot of the multivariable logistic regression analysis to identify the independent predictors of intraoperative major blood loss in brain AVM microsurgery. Red, significant factor; Blue, non-significant factor.

According to the estimated blood loss from the anesthesia case record, of 131 patients collected in this study, 50 (38%) experienced the intraoperative major blood loss. In order to validate the objectivity and accuracy of the subgroups in this study. The relative changes of intraoperative and perioperative Hb / Hct were compared between the major and the non-major blood loss cohort (Figure 2). Compared with the non-major blood loss cohort, the statistical results show that the relative intraoperative changes of Hb and Hct in the major blood loss cohort were greater (ΔHb_intraop._, Major vs. Non-major, 37.05 vs. 16.61%, ****p < 0.0001; ΔHct_intraop._, Major vs. Non-major, 38.01 vs. 16.61%, ****p < 0.0001). Similarly, the relative perioperative changes of Hb and Hct in the major blood loss cohort were also greater (ΔHb_periop._, Major vs. Non-major, 27.95 vs. 17.67%, ***p = 0.0003; ΔHct_periop._, Major vs. Non-major, 29.62 vs. 18.78, ***p = 0.0002). Furthermore, other parameters related to intraoperative blood loss were also compared between the two cohorts, including crystalloid fluid replenishment, colloid fluid replenishment, plasma replenishment, autologous blood intake, suspended red blood cell intake, single donor platelet intake, coagulation factor intake and the intraoperative urine output (Supplementary Figure 1). These results demonstrated that the blood loss amount is significantly larger in the major blood loss cohort, which can confirm the accuracy and objectivity of our grouping methodology.

### 3.2 Study population characteristics

The demographic and AVM characteristics in the major and non-major blood loss cohorts are described in Table 1. Overall, the male/female ratio was approximately 2:1, and the average patient age was 35 years (range 7–62 years).

**TABLE 1:** Demographic and clinical characteristics of AVM patients with/without intraoperative major blood loss^a^.

Characteristic	Major blood loss cohort (*n* = 50)	Non-major blood loss cohort (*n* = 81)	*p*-value
Age, mean (SD), year	34 (16)	35 (17)	0.69
Female sex	20 (40)	24 (30)	0.37
AVM nidus location			0.22
Frontal	12 (24)	23 (28)	
Parietal	14 (28)	12 (15)	
Temporal	9 (18)	20 (25)	
Occipital	6 (12)	16 (20)	
Deep (BG, IC, CC, ventricle)	3 (6)	1 (1)	
Cerebellum	6 (12)	9 (11)	
Associated aneurysm	6 (12)	7 (9)	0.75
Supplemented SM score			0.005
≤ 6	32 (64)	69 (85)	
> 6	18 (36)	12 (15)	
Rupture	19 (38)	54 (67)	**0.001**
Clinical presentation			0.07
Seizure	15 (30)	9 (11)	
Headache	22 (44)	44 (54)	
Neurological deficits	5 (10)	15 (19)	
Incidental	3 (6)	3 (4)	
Other	5 (10)	10 (12)	
Eloquence	9 (18)	14 (17)	0.92
Size, cm			**< 0.001**
< 3	6 (12)	48 (59)	
3–6	24 (48)	28 (35)	
> 6	20 (39)	5 (6)	
Deep drainage	22 (44)	22 (27)	**0.04**
Nidus diffuse	24 (48)	23 (28)	**0.02**
Nartery, mean (SD)	3.2 (1.2)	2.4 (1.1)	**0.001**
Nvein, mean (SD)	1.5 (0.8)	1.1 (0.3)	**< 0.001**
Vmax, mean (SD), mm	9.8 (4.5)	5.3 (5.8)	**< 0.001**
bAFT, mean (SD), sec	3.2 (0.7)	3.0 (0.7)	0.23
Preop embolization	33 (66)	21 (26)	**< 0.001**
Preop mRS, mean (SD)	1.5 (1.0)	1.6 (1.1)	0.42

BG, basal ganglia; CC, corpus callosum; IC, internal capsule; mRS, modified Rankin Scale score; Preop, Preoperative; SD, standard deviation; SM, Spetzler-Martin; Nartery, number of feeding arteries; Nvein, number of draining veins; Vmax, maximum diameter of draining veins; bAFT, brain AVM flushing time. ^a^Data are presented as no. (%) unless otherwise indicated. Values are bolded to highlight data with a statistical difference of *p* < 0.05.

Brain AVMs of the major blood loss cohort had higher supplemented SM score (36% with score > 6 vs. 15%, *p* = 0.005). In detail, these lesions were less likely to be ruptured prior to treatment (38 vs. 64%, *p* = 0.001) and tended to fall into larger AVM categories (87% with diameter > 3 cm vs. 41%, *p* < 0.001). Furthermore, these lesions were more likely to have deep venous drainage (44% with deep drainage vs. 27%, *p* = 0.04) and be diffuse (48% with nidus diffuse vs. 28%, *p* = 0.02). With respect to angio-architectural characteristics, lesions in the major blood loss cohort had more feeding arteries (3.2 ± 1.2 vs. 2.4 ± 1.1, *p* = 0.001) and more draining veins (1.5 ± 0.8 vs. 1.1 ± 0.3, *p* < 0.001). The maximum diameter of draining veins was also larger (9.8 ± 4.5 mm vs. 5.3 ± 5.8 mm, *p* < 0.001). And more patients in the major blood loss cohort were treated with endovascular embolization prior to resection (66 vs. 26%, *p* < 0.001). Nevertheless, there was no difference found between the two cohorts with respect to bAFT and the location of AVM nidus. The surgical characteristics in both cohorts were also not significantly different (Supplementary Table 1).

### 3.3 Predictors of intraoperative major blood loss

Important demographic and clinical factors for intraoperative major blood loss during brain AVM microsurgery were included in the univariate logistic regression (Supplementary Table 2). To avoid important factors being omitted, risk factors found on the univariate analysis to have *p*-values < 0.2 were included in a multivariate stepwise logistic regression.

**FIGURE 3:**
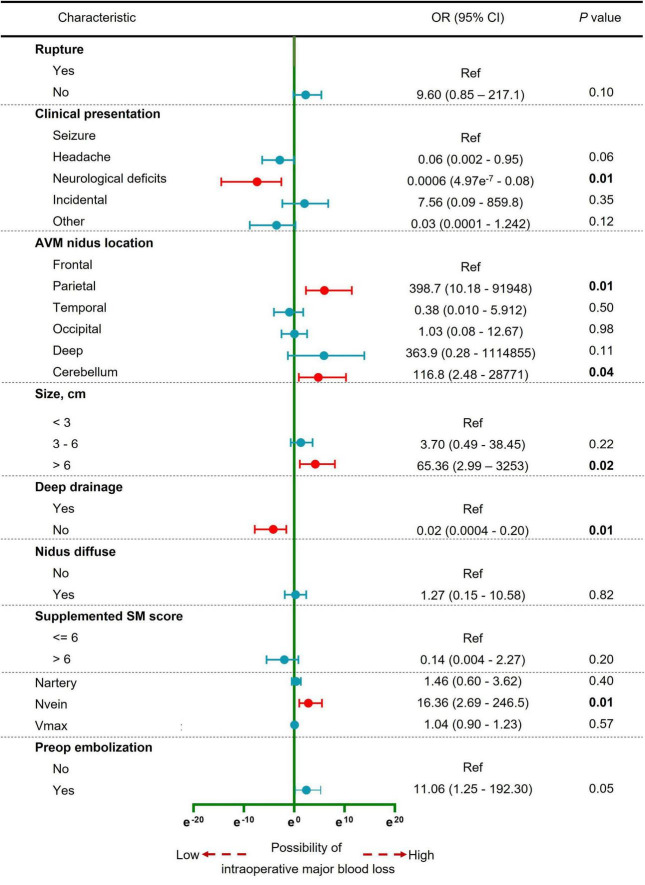
Forest plot of the multivariable logistic regression analysis to identify the independent predictors of intraoperative major blood loss in brain AVM microsurgery. Red, significant factor; Blue, non-significant factor.

**FIGURE 4:**
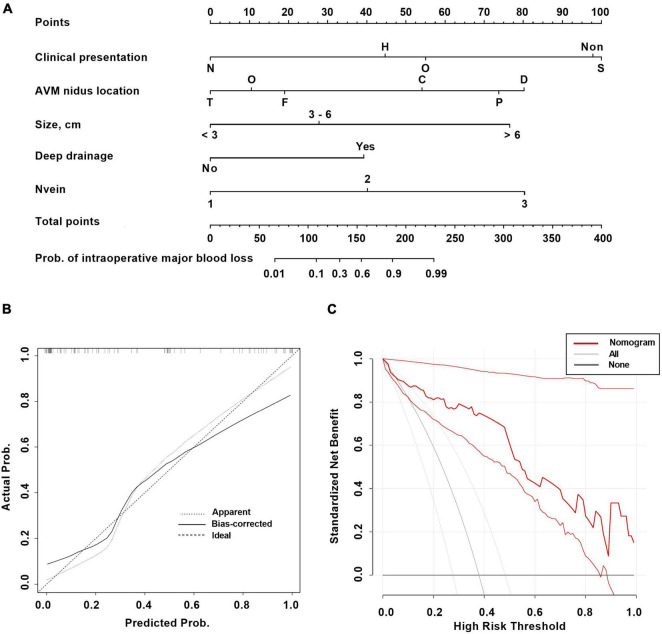
The nomogram **(A)** and calibration curve **(B)** and decision curve **(C)** based on the multivariable logistic regression analysis for predicting intraoperative major blood loss in brain AVM microsurgery. Clinical presentation: S, seizure; H, headache; N, neurological deficits; Non, incidental; O, other. AVM nidus location: F, frontal; P, parietal; T, temporal; O, occipital; D, deep; C, cerebellum.

A total of 11 variables were included in multivariate analysis (Figure 3). And 5 variables were found to be significantly associated with intraoperative major blood loss in brain AVM microsurgery: clinical presentation (neurological deficits vs. seizure, OR 0.0006, 95% CI 4.97e-7–0.08), AVM nidus location (parietal vs. frontal, OR 398.7, 95% CI 10.18–91948; cerebellum vs. frontal, OR 116.8, 95% CI 2.48–28771), AVM size (> 6 cm vs. < 3 cm, OR 65.36, 95% CI 2.99–3253), deep drainage (No vs. Yes, OR 0.02, 95% CI 0.0004–0.20), and the number of draining veins (OR 16.36, 95% CI 2.69–246.5). The AUC is 0.92 (95% CI 0.87–0.98, *p* < 0.001), indicating excellent predictive model performance. Additionally, we constructed a nomogram to estimate the probability of intraoperative blood loss in Figure 4. The calibration curve showed good agreement between observations and the nomogram for predicting the probability of intraoperative major blood loss. The decision curve indicated that application of the nomogram to predict intraoperative major blood loss is beneficial at all threshold probabilities. Further internal data validation still suggested a satisfied performance of this predictive model (see Supplementary Figure 2).

### 3.4 Surgical outcome

The impact of intraoperative major blood loss on patient outcomes was shown in Table 2. In term of peri-operation period, length of surgery (*p* < 0.001) and postoperative length of stay (*p* = 0.002) were both shorter in the intraoperative non-major blood loss cohort. Moreover, the risk of severe surgical complications was higher in the intraoperative major blood loss cohort (*p* = 0.005). In term of the prognosis, no significant differences were found for the rate of residual AVM. Nevertheless, the proportion of mRS score > 2 at the latest follow-up was higher in the major blood loss cohort (*p* = 0.03), suggesting that intraoperative major blood loss is associated with a poorer prognosis.

**TABLE 2:** Outcomes of AVM patients with and without intraoperative major blood loss^a^.

Characteristic	Major blood loss cohort (*n* = 50)	Non-major blood loss cohort (*n* = 81)	*p*-value
Length of surgery, mean (SD), min	515.4 (161.4)	284.5 (121.5)	**< 0.001**
Postop hospitalization days, mean (SD), d	16.4 (11.3)	11.0 (4.1)	**0.002**
Severe surgical complications^b^	15 (30)	5 (6)	**< 0.001**
Complete resection	48 (96)	78 (96)	0.70
Latest follow-up mRS score > 2	9 (18)	4 (5)	**0.03**
Follow-up duration, mean (SD), m	34.9 (16.5)	28.1 (17.3)	0.07

Postop, Postoperative. ^a^Data are presented as no. (%) unless otherwise indicated. ^b^Severe complications included severe pneumonia, severe cerebral edema and hydrocephalus, brain herniation, massive cerebral infarction, circulatory and respiratory failure, massive blood accumulation in the operative area and postoperative subdural (epidural) hematoma. Values are bolded to highlight data with a statistical difference of *p* < 0.05.

### 3.5 BW coated bipolar electrocoagulation in AVM microsurgery

**FIGURE 5:**
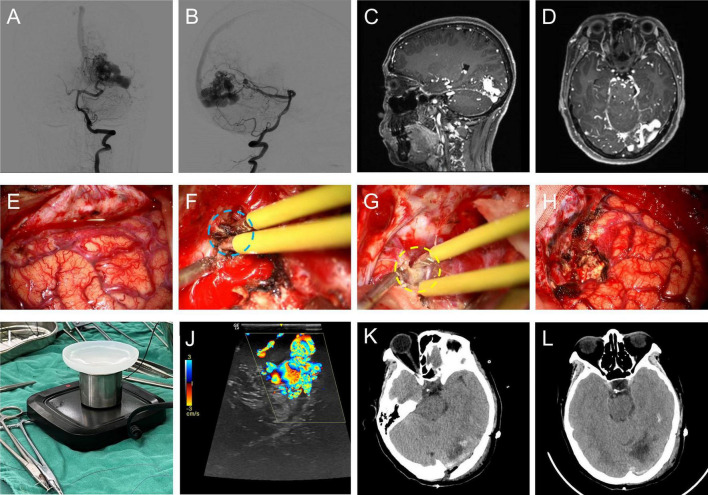
A typical case of brain AVM microsurgery using bone-wax coated bipolar electrocoagulation for hemostasis intraoperatively. Anterior **(A)** / Lateral **(B)** DSA image of the left vertebral artery; sagittal **(C)** / horizontal **(D)** MRA image of the brain AVM; **(E)** the exposed occipital cortex; intraoperative hemostasis using conventional bipolar electrocoagulation **(F)** / bone-wax coated bipolar electrocoagulation **(G)**; **(H)** occipital cortex after resection of the brain AVM lesion; **(I)** intraoperative photograph of the bone-wax coated bipolar electrocoagulation application device; **(J)** intraoperative ultrasound exploration of the brain AVM lesion; CT image of the operated area on the first day of postoperative review **(K)** / on the fifth day of postoperative review **(L)**.

A typical case for the use of BW coated bipolar electrocoagulation in brain AVM microsurgery was illustrated in Figure 5. As shown in Figures 5F, G, BW coated bipolar electrocoagulation caused less carbonization of the vascular structures till complete hemostasis compared with conventional bipolar electrocoagulation. In addition, histopathological characterization of different electrocoagulation on the vasculature of the nidus of brain AVMs showed that BW coated bipolar electrocoagulation caused less damage to the vessels, which mainly relied on the dual hemostatic mechanism (thermal and mechanical) (Supplementary Figure 3). BW for sealing the vascular wall can be observed around the wall and in the lumen of the vessels. Transmission electron microscopy scans further verified that BW-coated bipolar electrocoagulation caused less damage (see Supplementary Figure 4). Furthermore, the detailed intraoperative hemostatic efficiency of BW coated bipolar electrocoagulation was shown in Video.^1^ As mentioned above, this novel hemostatic method can be easily, safely, and efficiently used for microsurgery of brain AVMs. However, the hemostatic effect of BW coated bipolar electrocoagulation in microsurgery of brain AVMs still needs to be fully investigated.

## 4 Discussion

Intraoperative blood loss is an important factor required careful consideration for surgical planning in brain AVM microsurgery. Meanwhile, as mentioned above, the intrinsic characteristics of brain AVMs make it highly susceptible to ferocious bleeding during surgery, and the bleeding is not easily controlled. SM grading system and its supplemented grading system is widely used to evaluate the surgical risk of brain AVM microsurgery (15, 16). However, these grading systems may not be able to assess the risk of intraoperative blood major loss accurately. Factors such as eloquence of adjacent brain and age do not seem to be related to intraoperative blood loss. Therefore, the study of intraoperative blood loss fits the surgical difficulties and has significant clinical implications.

### 4.1 Statement of principal findings

First, we compared the parameters related to intraoperative blood loss in the major and non-major blood loss cohorts, which provided compelling evidence to confirm the accuracy and objectivity of our grouping methodology. Based on this, a multivariate logistic regression model was constructed to predict the risk of intraoperative major blood loss in brain AVM microsurgery. To facilitate the application for clinicians, the predictive nomogram was also constructed to present results. This predictive model included 5 variables, which were clinical presentation, location of AVM nidus, AVM size, venous drainage pattern, and number of draining veins. With this nomogram, managements for intraoperative major blood loss can be prepared in advance to facilitate the successful completion of surgeries. Such surgical managements include autologous blood transfusion, adequate preoperative blood preparation, more careful surgical operations and etc. Furthermore, our results revealed that intraoperative major blood loss has a detrimental effect on both the perioperative period and the prognosis of the patient. To cope with the challenge of intraoperative ferocious bleeding, BW coated bipolar electrocoagulation was applied. In addition to more efficient hemostasis, this novel hemostatic technology caused less damage to the electro-coagulated vessels. To our knowledge, this study is the first systematic study on the intraoperative blood loss in brain AVM microsurgery. The study didn’t only propose a simple model for neurosurgeons to predict the possibility of intraoperative major blood loss in brain AVM microsurgery and make better surgical planning in advance, but also provided a safer and efficient hemostatic method for intraoperative bleeding that is difficult to control.

### 4.2 Relation to other studies

As far as we know, the intraoperative blood loss in brain AVM microsurgery is a very relevant topic that still finds little space in the literature. A retrospective analysis by Donzelli et al. (3) demonstrated that intraoperative blood loss in brain AVM microsurgery was related to patient age and the AVM size. Probably due to the different study schemes, patient age was not significantly associated with the intraoperative major blood loss in our study. We analyzed the risk factors for subgroups of intraoperative blood loss rather than absolute values of intraoperative blood loss. In our perspective and based on previous researches (10), compared with absolute quantitative analysis, subgroup analysis can reduce bias in research data, demonstrate the detrimental effects of intraoperative blood loss, and more appropriately reflect the clinical value of the study. Similar to the SM grading, larger AVM size and deep venous drainage pattern was related to intraoperative major blood loss. Intriguingly, the patient clinical presentation was an independent risk factor of intraoperative major blood loss, suggesting a relation between the presentation and the intrinsic characteristics of brain AVM. Arterial steal phenomenon and venous outflow disruption may contribute to the etiology of seizure development (17). Previous studies have identified an association between seizure and venous outflow obstruction, venous varix and long draining veins (18, 19). These angio-architectural features imply that draining veins are susceptible to inadvertent injury during surgery, leading to stenosis or occlusion, which can result in severe intraoperative bleeding. Moreover, venous outflow disruption can cause tissue congestion and accompanied peri-AVM edema (19, 20), significantly increasing the risk of bleeding during the procedure. From a hemodynamic perspective, seizures have been linked to more pronounced hemodynamic alterations in brain AVM patients, as measured by blood oxygenation level–dependent cerebrovascular reactivity imaging (17). The presentation of seizures in brain AVM patients is associated with gliosis caused by chronic hypoxemia, which is primarily linked to severe steal phenomenon. Consequently, seizures may indicate a larger affected area and more complex hemodynamics, leading to more difficult surgery. In contrast, the absence of venous stenosis may contribute to headache symptomatology in unruptured brain AVM patients (21). And for ruptured brain AVM patients, hematoma can provide reasonable dissection planes to facilitate the lesion resection (9). Choi et al.’s (22) analysis found that patients who exhibited neurological deficits were also less likely associated with venous stenosis. These findings suggest that clinical manifestations such as headache and neurologic deficits are less likely to predict intraoperative major blood loss. Nevertheless, Other clinical manifestations remain to be further studied systematically. In term of the location, we found that deep-seated AVMs were at the highest risk of intraoperative major blood loss. Therefore, stereotactic radiosurgery and endovascular embolization are more often applied to this type of lesions, which are less accessible for microsurgery (23). The number of draining veins is an independent risk factor, which may be explained by the fact that the number of draining veins reflects more richer blood supply of the brain AVM nidus and the greater susceptibility of the draining vein injury by improper manipulation.

Notably, this study found a higher rate of combined embolization in intraoperative major blood loss cohort. However, can it be argued that preoperative embolization not only doesn’t reduce intraoperative blood loss, but rather increases it? The answer is no. Because the characteristics of brain AVMs treated with combined preoperative embolization are often more complex (see Supplementary Table 3), which would be difficult for microsurgical resection alone. Over the past 20 to 30 years, endovascular embolization has gradually been accepted as an adjunct to microsurgical resection, with the objectives of reducing the high-risk hemodynamic features of brain AVMs (24, 25). Preoperative embolization is intended to decrease the intraoperative blood loss by eliminating not readily accessible feeding arteries, occluding flow-related aneurysms, and reducing nide volume (3, 26). However, the role of preoperative embolization in the subsequent microsurgery is still controversial. The possible reasons include the unbalanced selection of patient population in different studies, heterogeneity in treatment goals, unreasonable study design, and inconsistent observation indicators (3, 27, 28). In a newly published study, researchers used a relative objective quantitative technique to assess the impact of preoperative embolization on intraoperative blood loss (29). And they found that patients with larger brain AVMs who underwent preoperative embolization had comparable intraoperative blood loss to those with smaller brain AVMs undergoing only surgical treatment, which addressed the value of preoperative embolization on brain AVMs surgical resection. More clinical trials are needed to objectively assess the role of preoperative embolization.

Many new techniques are emerging for complex brain AVMs microsurgery, including mixed reality (30), intraoperative hemodynamic monitoring by laser speckle contrast imaging (31), and etc. In this study, we introduced an efficient hemostatic method, BW coated bipolar electrocoagulation, in brain AVMs microsurgery. Histological abnormalities in the vascular structure contribute largely to the difficulty of hemostasis in brain AVM microsurgery. Previous studies and findings in our study have reveled structural imperfectness and immaturity of the AVM vascular wall (8, 32, 33). Moreover, pericytes and vascular smooth muscle cells are associated with and may contribute to vascular fragility and hemodynamic changes in brain AVMs (34, 35). The aberrant smooth muscle layers and the enlarged lumen make the AVM vessels resistant to electrocoagulation contraction. Based on the mechanical and thermal dual hemostatic mechanism, BW coated bipolar electrocoagulation has been proven to be more-efficient for hemostasis (12). Here, we presented the initial application condition of this novel hemostatic technique in brain AVM microsurgery. BW coated bipolar electrocoagulation did not only exert efficient hemostatic effect, but also reduced the damage to the vascular structure. Regarding the safety of this hemostatic method, almost all excessive BW used for electrocoagulation leaves the cranial cavity either by suction or with the lesion resection. And no complications related to BW use occurred in our patients. Besides, BW coating can also alleviate the damage caused by electrocoagulation to the forceps themselves, thereby extending the service life of the forceps. The initial protective coating of bipolar electrocoagulation forceps is susceptible to damage due to the high frequency of use, resulting in decreased efficiency of electrocoagulation hemostasis and adhesion damage to surrounding tissues. Above all, BW coated bipolar electrocoagulation is a practical and efficient hemostatic method in brain AVM microsurgery, which can be applied to control fierce bleeding.

### 4.3 Limitations

Our study had several limitations. First, sample size is a nonnegligible factor limiting this study. And the study was a single-center retrospective study and therefore is subject to bias. Although internal data cross validation was performed, external data cross validation for model prediction accuracy analysis of intraoperative major blood loss is still lacking. Hence, the model extrapolation has some limitations. Second, the introduction of the impact of BW bipolar electrocoagulation on brain AVM microsurgery is relative superficial in this study, and further broader and more comprehensive studies are needed.

## 5 Conclusion

Five variables are included in the predictive nomogram: clinical manifestation, AVM lesion location, AVM size, venous drainage pattern, and number of draining veins. Neurosurgeons can predict the risk of intraoperative major blood loss in brain AVM microsurgery preoperatively by this nomogram. And results indicates that the intraoperative major blood loss is associated with poor patient outcomes. This study also shows that BW coated bipolar electrocoagulation can be applied efficiently and safely in brain AVM microsurgery, which needs to be further verified.

## Data availability statement

The original contributions presented in this study are included in this article/Supplementary material, further inquiries can be directed to the corresponding authors.

## Ethics statement

The studies involving humans were approved by the Local Ethics Committee of Zhongnan Hospital of Wuhan University. The studies were conducted in accordance with the local legislation and institutional requirements. The ethics committee/institutional review board waived the requirement of written informed consent for participation from the participants or the participants’ legal guardians/next of kin because due to the low risk of a privacy breach and the retrospective nature of the study, a waiver for patient consent was granted.

## Author contributions

JS: Conceptualization, Data curation, Formal analysis, Investigation, Methodology, Writing – original draft, Writing – review & editing. SX: Data curation, Investigation, Validation, Writing – original draft. YF: Conceptualization, Data curation, Investigation, Methodology, Validation, Writing – original draft. WW: Formal analysis, Writing – review & editing. YZ: Data curation, Investigation, Writing – original draft. WX: Data curation, Methodology, Writing – original draft. WZ: Methodology, Validation, Writing – original draft. TZ: Conceptualization, Data curation, Funding acquisition, Supervision, Writing – review & editing. HP: Conceptualization, Funding acquisition, Methodology, Supervision, Writing – review & editing. JC: Conceptualization, Funding acquisition, Supervision, Validation, Writing – review & editing.

## Footnotes

1
http://vimeo.com/796535477
