# Supplementary material for: Predicting intraoperative major blood loss in microsurgery for brain arteriovenous malformations
Source: Front Med (Lausanne). 2024 Aug 7;11:1446088. doi: 10.3389/fmed.2024.1446088 (PMC11335480; doi:10.3389/fmed.2024.1446088)
Supplement: Supplementary file 1 [file Data_Sheet_1.docx]

Supplementary Material

# Supplementary Figures and Tables

##
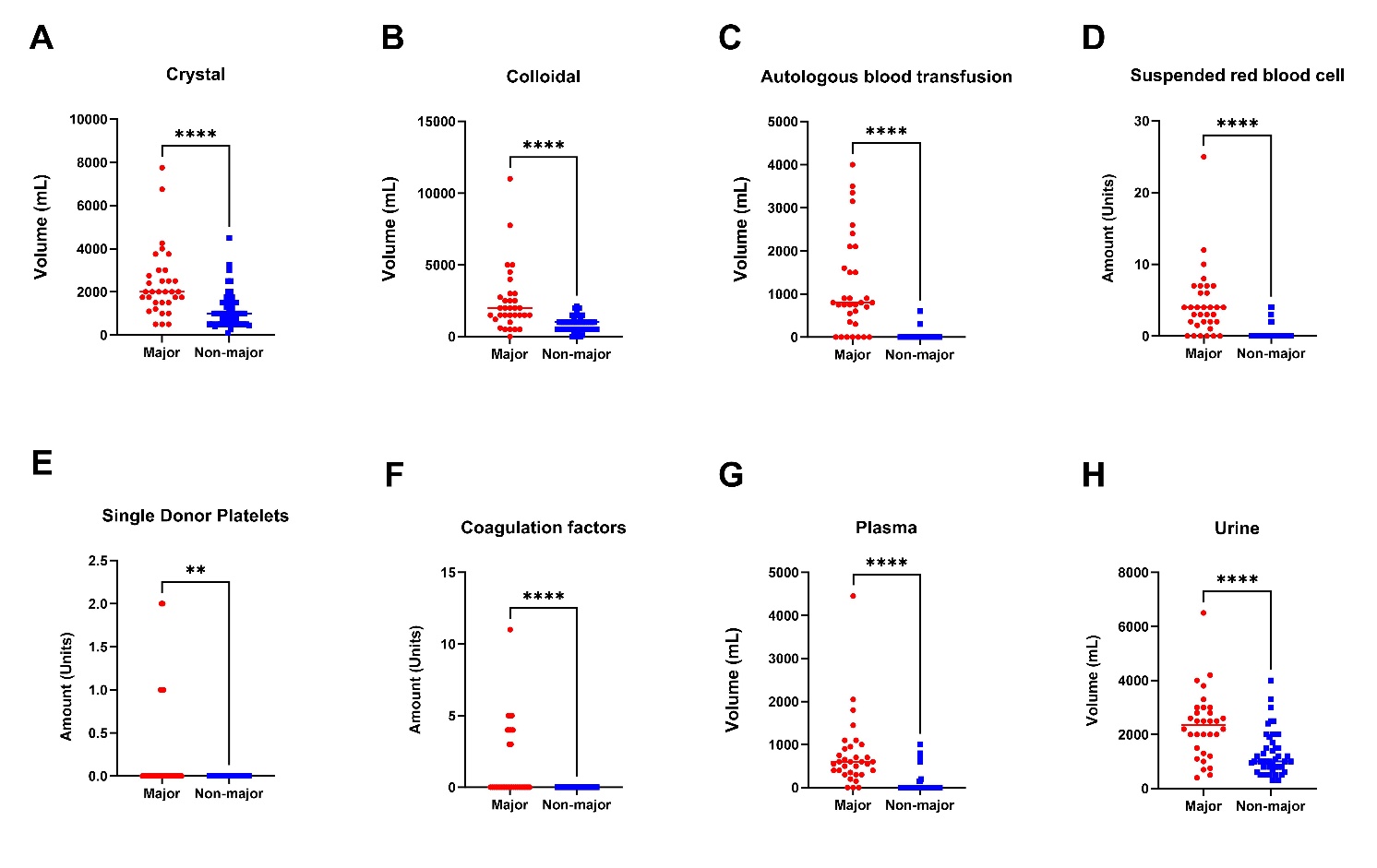
Supplementary Figures

**Supplementary Figure 1.** Intraoperative crystalloid fluid replenishment (**A**), colloid fluid replenishment (**B**), autologous blood transfusion (**C**), suspended red blood cell intake (**D**), single donor platelet intake (**E**), coagulation factor intake (**F**), plasma intake (**G**), and urine output (**H**) of the patients in both groups. n = 50 in the major blood loss group and n = 81 in the non-major blood loss group. Two-tailed unpaired *t*-test, ** *p* < 0.01, **** *p* < 0.0001.


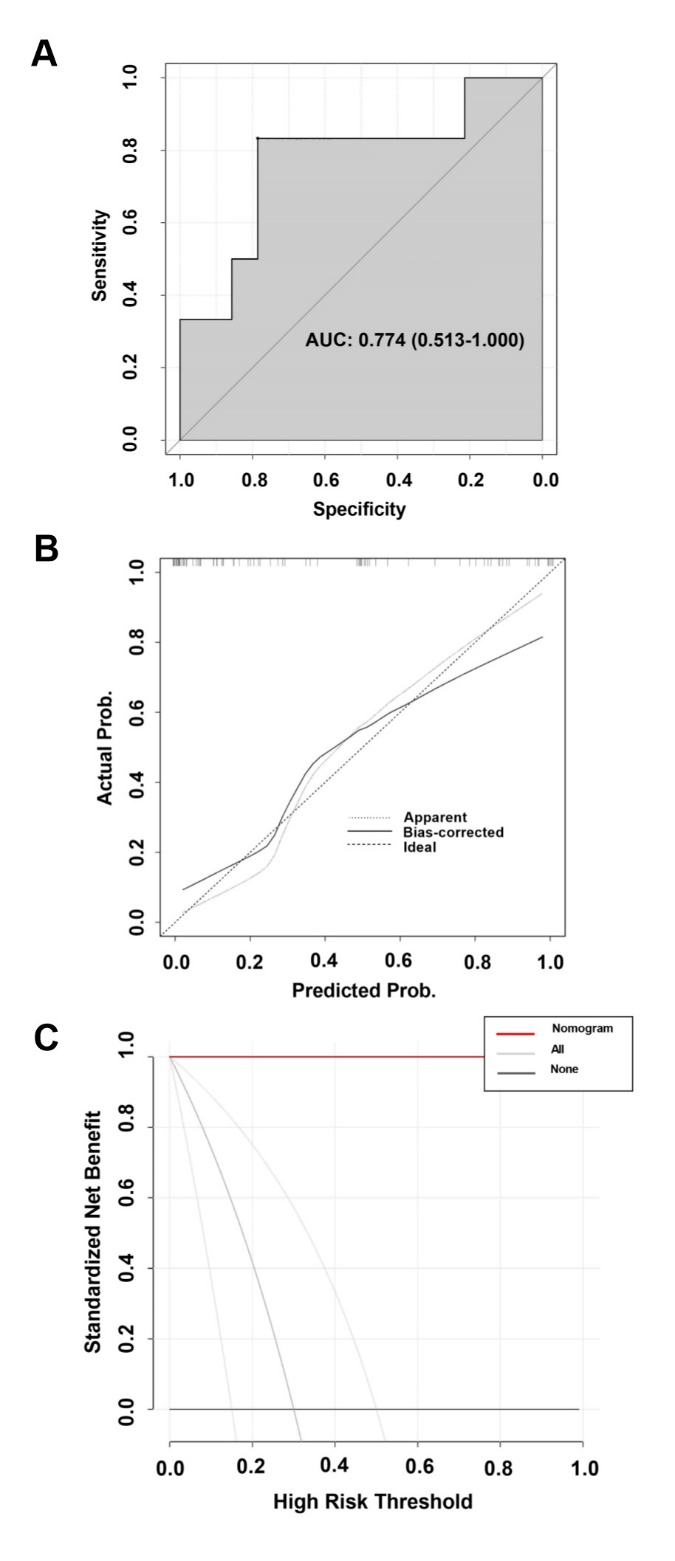
**Supplementary Figure 2.** Internal data validation for model prediction accuracy analysis of intraoperative major blood loss in brain AVM microsurgery. The ROC curve (**A**), calibration curve (**B**) and decision curve (**C**) of the validation dataset. The validation dataset included the data for patients treated in our center who underwent microsurgical resection of brain AVMs during the period between May 2023 and January 2024 (n = 20).


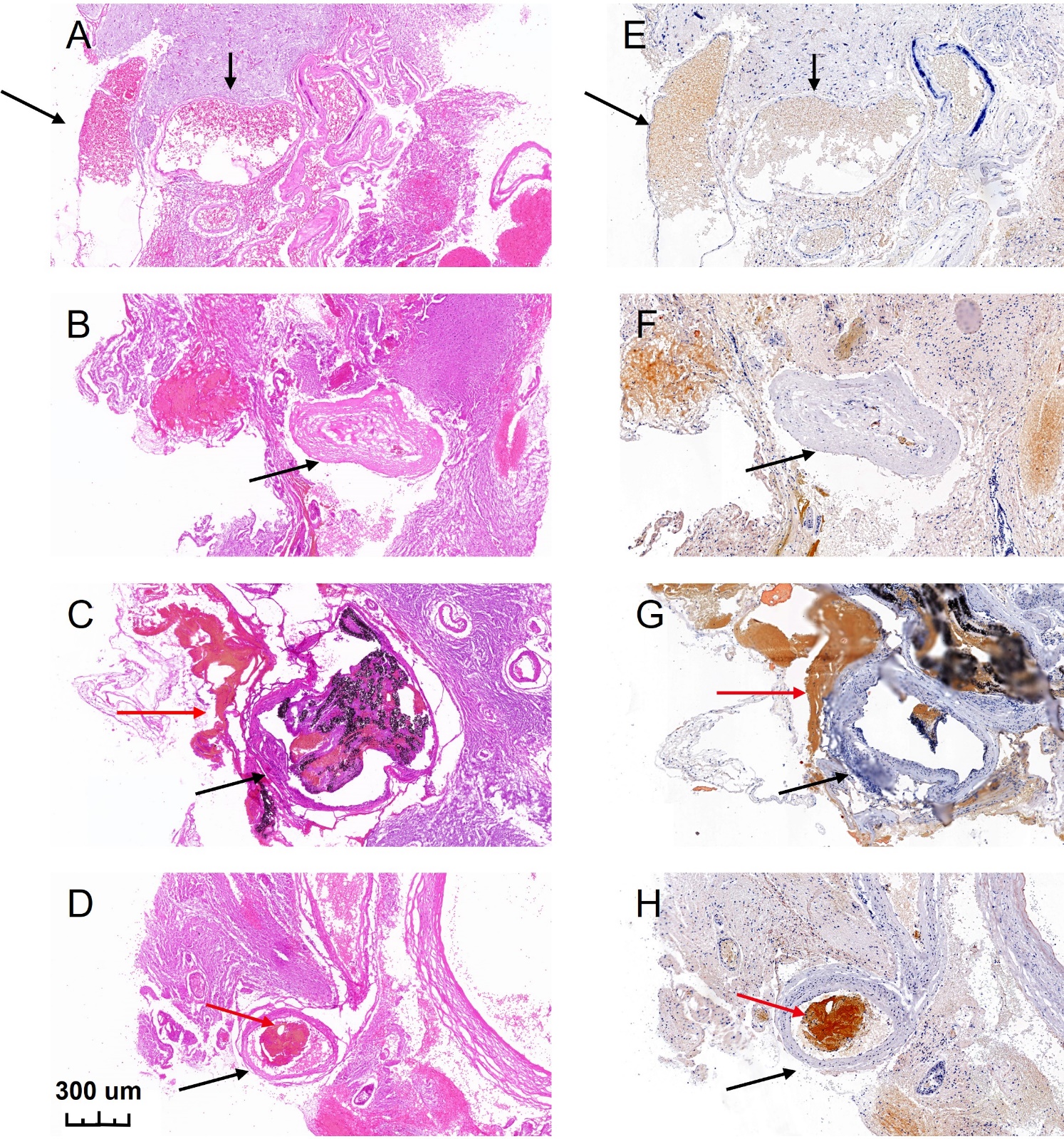


**Supplementary Figure 3.** The histopathological features of brain AVM nidus vessels electro-coagulated by different bipolar electrocoagulation. (**A, E**) the AVM nidus vessel; (**B, F**) the AVM nidus vessel electro-coagulated by conventional electrocoagulation; (**C, D, G, H**) the AVM nidus vessel electro-coagulated by BW coated bipolar electrocoagulation. (**A - D**) hematoxylin-eosin staining; (**E - H**)
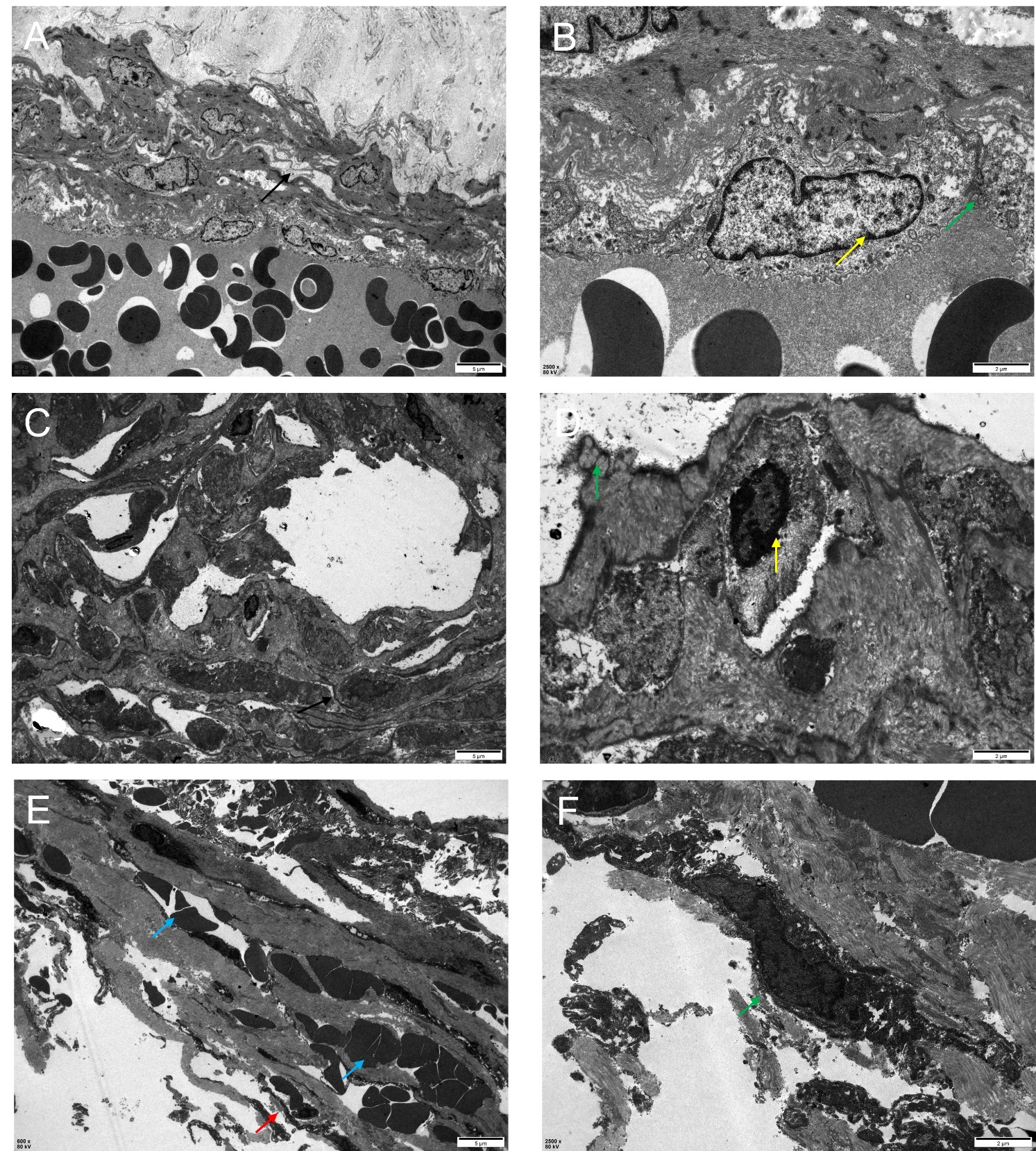
oil red O staining. Black arrow, nidus vessel; red arrow, residual BW. Magnification: 12X.

**Supplementary Figure 4.** Transmission electron microscopy of brain AVM nidus vessels after electrocoagulation 400 by different bipolar electrocoagulation. The original state of brain AVM nidus vessels without electrocoagulation (**A - B**); the state of brain AVM nidus vessels with conventional bipolar electrocoagulation (**C - D**) / the bone-wax coated bipolar electrocoagulation (**E - F**). **A, C, E,** the low magnification; **B, D, F,** the high magnification. (**A**) the basement membrane of the vessel wall is continuous, and the intercellular space in the vascular medium membrane increased, as shown by the black arrow. (**B**) The chromatin boundary set in the vascular endothelial nucleus is seen, as shown by the yellow arrow. And the endothelium of the blood vessels is smooth and the endothelial cells are tightly connected, as shown by the green arrow. (**C**) The basal membrane of the blood vessel wall was broken, the cell morphology in the middle membrane of the blood vessel was changed, the cell boundary was unclear, no organelle structure was found in the cell, and the cell arrangement was seriously disordered, as shown by the black arrow. (**D**) Some vascular endothelial nuclei have chromatin border sets, and no other organelle structures can be seen in the cells, as shown by the yellow arrow. Vascular endothelial smoothness is reduced, some endothelial cells are tightly connected and open, and local prominences can be seen, as shown by the green arrow. (**E**) Local rupture and shedding of the intima can be seen, as shown by the red arrow. The vascular media is locally stratified, the intercellular space is enlarged and filled with red blood cells, as shown by the blue arrow. (**F**) Vascular endothelial smoothness is reduced, showing incomplete vascular endothelium, local endothelial cells are tightly connected and open, endothelial nucleus chromatin edge set, and no organelle structure is seen inside the cell, as shown by the green arrow. **A – F,** magnifications are at the bottom right corner of each figure.

## Supplementary Tables

**Supplementary Table 1**

Surgical characteristics of AVM patients with and without intraoperative major blood loss^a^.

| Characteristic | Major blood loss cohort  (n = 50) | Non-major blood loss cohort  (n = 81) | p value |
| --- | --- | --- | --- |
| Coagulation abnormalities | 12 (24) | 23 (28) | 0.58 |
| ASA score > 2 | 14 (28) | 20 (25) | 0.67 |
| Surgeon |  |  | 0.73 |
| Dr. Chen | 30 (60) | 51 (63) |  |
| Other | 20 (40) | 30 (37) |  |
| Position |  |  | 0.49 |
| Supine | 33 (66) | 60 (74) |  |
| Lateral | 8 (16) | 6 (7) |  |
| Prone | 6 (12) | 10 (12) |  |
| Sitting | 3 (6) | 5 (6) |  |
| Emergency | 5 (10) | 11 (14) | 0.74 |

^a^Data are presented as no. (%) unless otherwise indicated.

**Supplementary Table 2**

Univariate analysis of risk factors for intraoperative major blood loss during brain AVM microsurgery.

| Characteristic | OR (95% CI) | p value |
| --- | --- | --- |
| Age, yr |  |  |
| < 20 | Ref |  |
| 20 - 40 | 0.83 (0.26 - 2.67) | 0.74 |
| > 40 | 0.78 (0.25 - 2.46) | 0.66 |
| Sex |  |  |
| Male | Ref |  |
| Female | 0.63 (0.30 – 1.33) | 0.22 |
| Rupture |  |  |
| No | Ref |  |
| Yes | 3.26 (1.58 – 6.90) | **0.02** |
| Clinical presentation |  |  |
| Seizure | Ref |  |
| Headache | 0.30 (0.11 - 0.78) | **0.02** |
| Neurological deficits | 0.20 (0.07 - 0.70) | **0.02** |
| Incidental | 0.30 (0.09 - 3.85) | 0.58 |
| Other | 0.60 (0.07 - 1.12) | **0.08** |
| AVM nidus location |  |  |
| Frontal | Ref |  |
| Parietal | 2.24 (0.80 – 6.47) | **0.13** |
| Temporal | 0.86 (0.30 – 2.46) | 0.78 |
| Occipital | 0.72 (0.21 - 2.27) | 0.58 |
| Deep | 5.75 (0.66 - 123.40) | **0.15** |
| Cerebellum | 1.28 (0.36 - 4.44) | 0.70 |
| Side |  |  |
| Left | Ref |  |
| Right | 1.04 (0.51 – 2.11) | 0.92 |
| Infratentorial | 0.89 (0.26 – 2.74) | 0.84 |
| Eloquence |  |  |
| Yes | Ref |  |
| No | 0.95 (0.38 - 2.47) | 0.92 |
| Associated aneurysm |  |  |
| Yes | Ref |  |
| No | 0.69 (0.22 – 2.78) | 0.53 |
| Size, cm |  |  |
| < 3 | Ref |  |
| 3- 6 | 6.86 (2.64 - 20.38) | **< 0.001** |
| > 6 | 32.00 (9.50 - 130.60) | **< 0.001** |
| Deep drainage |  |  |
| Yes | Ref |  |
| No | 0.47 (0.22 - 0.99) | **0.04** |
| Nidus diffuse |  |  |
| No | Ref |  |
| Yes | 0.43 (0.20 - 0.89) | **0.02** |
| Supplemented SM score |  |  |
| <= 6 | Ref |  |
| > 6 | 3.23 (1.41 – 7.67) | **0.006** |
| Nartery | 1.94 (1.29 - 3.06) | **0.002** |
| Nvein | 4.58 (1.89 - 13.39) | **0.002** |
| Vmax | 1.21 (1.08 - 1.37) | **0.002** |
| bAFT | 0.66 (0.33 – 1.28) | 0.23 |
| Preop embolization |  |  |
| No | Ref |  |
| Yes | 5.55 (2.62 – 12.21) | **< 0.001** |
| Preop mRS | 0.79 (0.20 – 2.76) | 0.72 |

SM, Spetzler-Martin; Nartery, Number of feeding arteries; Nvein, Number of draining veins; Vmax, Maximum diameter of draining veins; bAFT, brain AVM flushing time; Preop, Preoperative; mRS, the modified Rankin Scale.

**Supplementary Table 3**

Characteristics of AVMs treated by microsurgery with and without endovascular embolization^a^.

| Characteristic | Without endovascular embolization cohort  (n = 80) | With endovascular embolization cohort  (n = 51) | p value |
| --- | --- | --- | --- |
| Supplemented SM score |  |  | 0.16 |
| <= 6 | 65 (81) | 36 (71) |  |
| > 6 | 15 (19) | 15 (29) |  |
| Size, cm |  |  | **< 0.001** |
| < 3 | 42 (53) | 12 (24) |  |
| 3 - 6 | 33 (41) | 19 (37) |  |
| > 6 | 5 (6) | 20 (39) |  |
| Nartery, mean (SD) | 2.4 (0.1) | 3.2 (0.2) | **0.002** |
| Nvein, mean (SD) | 1.2 (0.1) | 1.4 (0.1) | 0.31 |

^a^Data are presented as no. (%) unless otherwise indicated.
